# Neural respiratory drive during maximal voluntary ventilation in individuals with hypertension: A case-control study

**DOI:** 10.1371/journal.pone.0305044

**Published:** 2024-06-11

**Authors:** Andressa V. S. O. N. Cavalcante, Jéssica Danielle Fonseca, Helen Rainara Araujo Cruz, Viviane Fabrícia Nascimento, João Pedro Santana Silva, Caio Alano Lins, Saint-Clair Gomes Bernardes Neto, Íllia Nadinne Dantas Lima

**Affiliations:** 1 Faculdade de Ciências da Saúde do Trairi (FACISA), Programa de Pós-Graduação em Ciências da Reabilitação, Santa Cruz, Rio Grande do Norte, Brazil; 2 Departamento de Fisioterapia, Universidade Federal do Rio Grande do Norte, Natal, Rio Grande do Norte, Brazil; 3 Faculdade de Ciências da Saúde do Trairi (FACISA), Laboratório de Motricidade e Fisiologia Humana, Santa Cruz, Rio Grande do Norte, Brazil; Instituto Nacional de Cardiologia Ignacio Chavez, MEXICO

## Abstract

Neural respiratory drive (NRD) is measured using a non-invasive recording of respiratory electromyographic signal. The parasternal intercostal muscle can assess the imbalance between the load and capacity of respiratory muscles and presents a similar pattern to diaphragmatic activity. We aimed to analyze the neural respiratory drive in seventeen individuals with hypertension during quite breathing and maximal voluntary ventilation (MVV) (103.9 ***±*** 5.89 vs. 122.6 *±* 5 l/min) in comparison with seventeen healthy subjects (46.5 ***±*** 2.5 vs. 46.4 *±* 2.4 years), respectively. The study protocol was composed of quite breathing during five minutes, maximum inspiratory pressure followed by maximal ventilatory ventilation (MVV) was recorded once for 15 seconds. Anthropometric measurements were collected, weight, height, waist, hip, and calf circumferences, waist-to-hip ratio (WHR), waist-to-height ratio (WHtR), BMI, and conicity index (CI). Differences between groups were analyzed using the unpaired *t*-test or Mann-Whitney test to determine the difference between groups and moments. A significance level of 5% (p<0,05) was adopted for all statistical analyses. The group of individuals with hypertension presented higher values when compared to the healthy group for neural respiratory drive (EMGpara% 17.9±1.3 vs. 13.1±0.8, p = 0.0006) and neural respiratory drive index (NRDi (AU) 320±25 vs. 205.7±15,p = 0.0004) during quiet breathing and maximal ventilatory ventilation (EMGpara% 29.3±2.7 vs. 18.3±0.8, p = 0.000, NRDi (AU) 3140±259.4 vs. 1886±73.1,p<0.0001), respectively. In conclusion, individuals with hypertension presented higher NRD during quiet breathing and maximal ventilatory ventilation when compared to healthy individuals.

## Introduction

Individuals with hypertension (including those without congestive heart failure) may present increased pulmonary artery pressure with right ventricular failure [1–3]. Also, this overload may decrease the arterial and venous blood flow to the lungs [4,5], reducing gas exchange and impairing respiratory function due to respiratory muscle weakness [6,7].

The respiratory pattern, control, and work of breathing contributes to blood pressure (BP) regulation [7–9]. Several studies have described that the respiratory cycle is synchronized with cardiovascular systems, through this sympathetic nerve discharge (SND). The concept of a central coupling of respiratory and sympathetic networks can maximize oxygen uptake and perfusion during rest, one should maximize minute ventilation and cardiac output. Hypertension influences the modulation of sympathetic nerve activity and this increase in blood pressure is made possible by the enhanced respiratory [10–14]. Also, individuals with hypertension may have lower forced vital capacity (FVC), forced expiratory volume in the first second (FEV1), and peak expiratory flow than healthy individuals [10]. Thus, this population may have impaired respiratory muscle strength, hampering lung ventilation and affecting BP regulation [9–15].

The parasternal intercostal muscle electromyography (EMGpara) has been established as a marker of NRD and assesses the imbalance between the load and capacity of respiratory muscles and presents a similar pattern to diaphragmatic activity with reproducible measurements. Based on this measurement the neural respiratory drive (NRD) is measured using a non-invasive recording of respiratory muscle electromyographic signal (EMG). [16–19].

In this regard, the present study aimed to assess the NRD in individuals with hypertension during quiet breathing (QB) and maximal voluntary ventilation (MVV) in comparison to healthy individuals. We hypothesize that NRD is influenced by arterial hypertension. The hypertensive individuals have a greater muscular activation at rest and during maximal ventilatory efforts compared to healthy individuals.

## Materials and methods

### Subjects and experimental design

This case-control study was approved by the research ethics committee (no. 3204568), followed the Declaration of Helsinki and all participants signed the informed consent form (written). The study was conducted using a convenience sample. The recruitment period for this study started on July 12, 2020 and ended in July 30, 2023, considering the pandemic pause period. To calculate the sample size, the G-power Software 3.1.9.4 version was used, considering the significance level (5%) and the power of 0.70.

Participants of hypertension group were included according to the following eligibility criteria: aged between 35 and 65 years; body mass index (BMI) classified as normal or overweight [20]; with controlled arterial hypertension diagnosis (systolic blood pressure [SBP] 130–139 and/or diastolic blood pressure [DBP] 85–89 were considered to be in the hypertension group, using anti-hypertensive medication [21]; and without diabetes, without acute myocardial infarction, unstable angina, severe arrhythmia, heart valve disease, decompensated heart failure, thrombophlebitis, pulmonary or systemic thromboembolism, cognitive impairment, diabetes, or orthopedic, rheumatological, or neurological disorders hampering assessments or causing risk. Self-reported healthy individuals matched for age were recruited for the control group.

### Anthropometric measurements and vital signs

Anthropometric measurements were collected according to the International Society for the Advancement of Kin anthropometry guidelines [22]. We collected body weight, height, waist, hip, and calf circumferences, waist-to-hip ratio (WHR), waist-to-height ratio (WHtR), BMI and conicity index (CI). Suprailiac, medial calf, subscapular, and triceps skinfolds [22] were measured and used in the Petroski equation [23] to estimate body fat.

The BMI was classified as underweight (< 18.5 kg/m^2^), normal weight (18.5 to 24.9 kg/m^2^), overweight (25 to 29.9 kg/m^2^), and obesity (≥ 30 kg/m^2^) [16,20]. Waist circumference ≥ 94 cm (men) or ≥ 80 cm (women) indicates an increased risk of metabolic complications associated with obesity, and ≥ 102 cm (men) or ≥ 88 cm (women) indicates a substantially increased risk [23]. WHR > 0.9 (men) or > 0.85 (women) indicates abdominal fat [23] and increased risk of metabolic complications (according to the World Health Organization) [24].

A single trained evaluator performed all measurements in duplicates, and the skinfold was measured on the right side of the body. The mean of the two measurements was considered for results. The precision of the error was calculated using the intra-evaluator technical error of measurement, which analyzes the standard deviation of alternative methods for several measurements. A variability of < 5% between skinfold measurements and < 1% for estimated value was considered acceptable precision.

The stadiometer that was used to measure the subjects’ height had a resolution of 1 cm and a range of 50–200 cm. A maximum capacity of 300 kg and a resolution of 50 g were features of the electronic scale (WELMY® model R110, Santa Bárbara d’ Oeste-SP, Brasil) that was calibrated every day. In order to calculate the subject’s body mass index, their weight was divided by the square of their height in meters. With a non-extensible measuring tape wrapped around the narrowest portion of the abdomen (or, if ambiguous, the midpoint between the inferior border of the tenth rib and the iliac crest) and the level of the greater trochanter, the circumferences of the waist and hips were measured in centimeters.

In addition to anthropometric variables, vital signs were measured before electrode placement during quiet breathing systolic blood pressure (SPB), diastolic blood pressure (DPB) (G-Tech®, model LA800, São Paulo, Brazil), heart rate (HR) and oxygen saturation (SpO2) (%) (Portable Pulse Oximeter, model UT-100, MD®, São Paulo, Brazil) and respiratory rate (RR) by the number of breaths per minute. These vital signs were measured again immediately after maximal voluntary ventilation. The participants were evaluated sitting in a comfortable research laboratory environment.

### Maximal voluntary ventilation

All participants performed MVV using the KoKo DigiDoser® spirometer (Longmont, USA). Participants were instructed to perform maximal ventilation by inhaling and exhaling as fast and deep as possible for 15 seconds [25], and the result (L/min) was extrapolated to estimate the air volume in 60 seconds to avoid prolonged hyperventilation [26]. An accuracy of ± 10% (± 15 L/min) was considered acceptable [27], and the predictive MVV was calculated using the formula: y = 37.5xFEV1 +15,8 [28].

### Electromyography

A trained evaluator collected the EMGpara of participants in sitting position (in a chair with back supported, arms placed on armrests and feet flat on the floor to reduce trunk postural activity) with the skin properly cleaned and prepared. Electrodes were placed on the right side in the second intercostal space three centimeters lateral to the sternum. Two silver/silver chloride electrodes were placed six-centimeter apart with a conductive gel [29], and a monopolar reference electrode (3.8 cm diameter of adhesive area and 1 cm of conductive area; Noraxon®, USA) was placed on the clavicle.

All electrodes were connected to the six-channel signal conditioning module (MCS 1000; EMG System do Brasil®). The 12-bit analog-to-digital converter (CAD, 12/36-60K) was connected to a computer software for signal capture and analysis (EMGLab v1.1, EMG System do Brasil Ltda) to acquire signals with a sampling frequency of 2 kHz. Signals were recorded using a differential amplifier (100 gain) and a band-pass filter (20 to 500 Hz).

**Fig 1:**
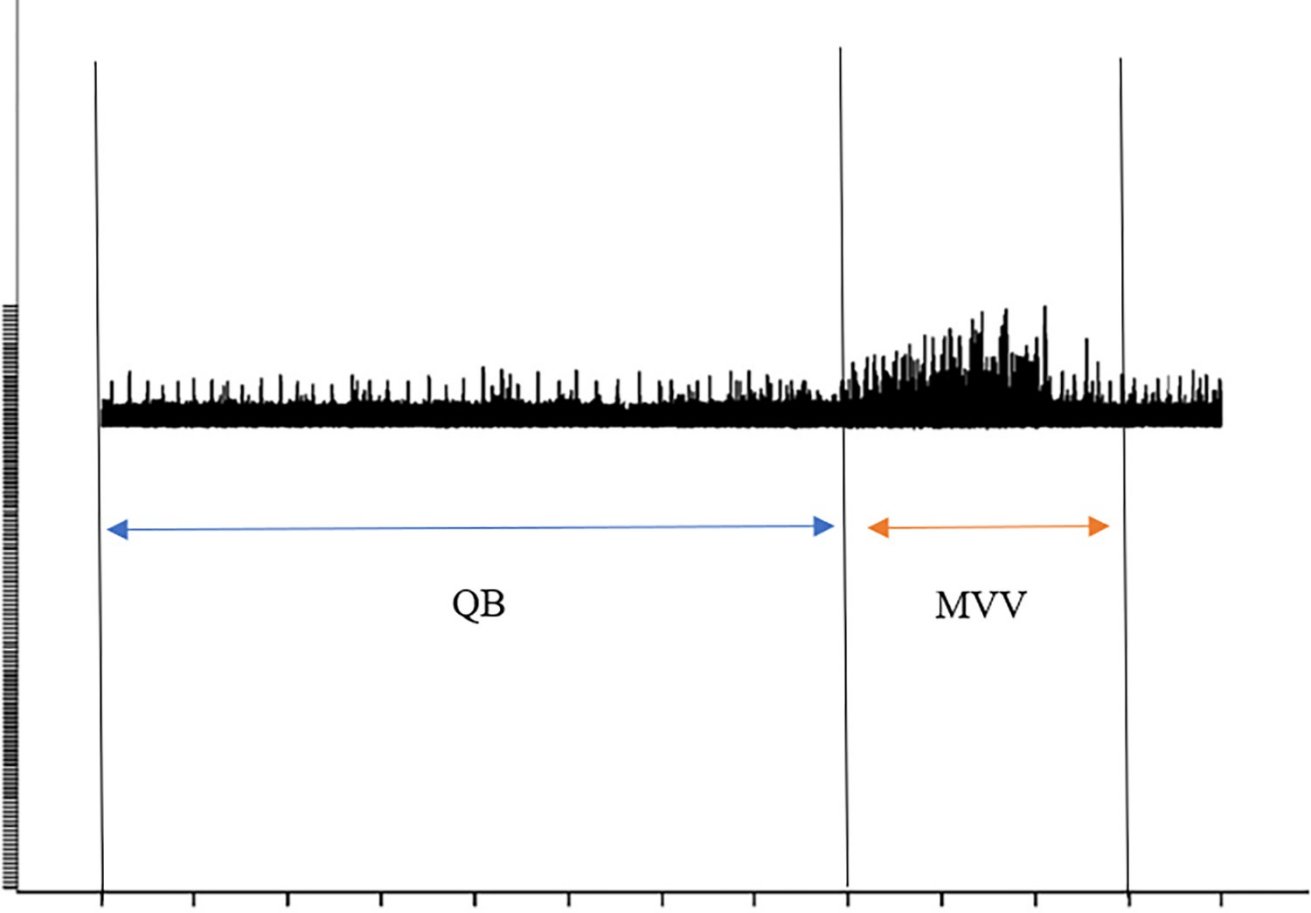
Representation of the raw parasternal intercostal muscle electromyography (EMGpara) (RMS) of hypertensive individual during quiet breathing and maximal voluntary ventilation. **X axis: Myoelectric activity, Y axis: Time (15 s).** QB—quiet breathing; MVV—maximal voluntary ventilation.

EMGpara was recorded during five minutes of quiet breathing without a mouthpiece. Next, the EMGpara was recorded during one maximal inspiratory effort to allow normalization of the resting signal to maximal EMGpara activity followed by MVV recorded once for 15 seconds. The highest EMGpara recorded during each MVV was identified and selected to ensure absence of artifact from external interference, crosstalk from other musculature or heart signal interference (Fig 1).

Data were presented as raw EMGpara and root mean square (RMS) using a moving average window of 50 ms. The mean peak RMS of EMGpara per breath was calculated at the final 60 seconds of each recording period. EMGpara was expressed as a raw RMS (μV) and percentage of the highest RMS of EMGpara during MIP (EMGpara%max). The NRD index (NRDi) was calculated as the product of EMGpara%max and respiratory rate in arbitrary units (AU) [30].

All procedures followed the recommendations of the Surface EMG for the Non-Invasive Assessment of Muscles project [29].

The collection procedures followed the order: anthropometric assessment, vitals signals, preparation for EMG, collection of electrical activity during 5 minutes of rest, followed by a maximum inspiratory effort, MVV and finally, immediately after MVV, the vitals signals again.

### Statistical analysis

Data were analyzed using the GraphPad Prism (version 7.0) (La Jolla, Ca, USA). The Shapiro-Wilk test verified data normality for quantitative variables. Data were presented as mean and standard deviation or median [interquartile range 25–75%] according to distribution. Differences between groups and moments (QB and MVV) were analyzed using the unpaired *t*-test or Mann-Whitney test was adopted to determine the difference between groups and QB (5 min) and MVV (15 sec). To effect-size and power analysis of the study the GPower software, version 3.1.9.2 (Kiel, Germany) was used. A significance level of 5% (p<0,05) was adopted for all statistical analyses.

## Results

Thirty-eight participants were screened. 34 participants were included in the study, including 17 subjects with diagnosis of hypertension (94.1% female) and 70.5% presented stage 1[31]. Participants in the hypertension group were non-smokers, and one consumed alcohol regularly. The control group consisted of 17 healthy subjects (86.6% female) without diagnosed cardiac or respiratory conditions. Despite repositioning the electrodes and repeating signal recording, four participants were excluded due to difficulties in collecting NRD signals. Sample characteristics and analyses of ventilatory capacity and EMGpara are presented in Tables 1 and 2.

**Table 1:** Anthropometric characteristics of the sample (Hypertension *vs*. Healthy).

Subjetcs (n)	Hypertension (n = 17)	Healthy (n = 17)	*p*
Age (Years)	46.5 ± 2.5	46.4 ± 2.4	.960
Weight (kg)	67.6 ± 2	66.8 ± 1.1	.729
Height (m)	1.5 ± 0.01	1.8 ± 0.02	.788
BMI (kg/m^2^)	28.9 [26.6–29.7]	27.4 [25.9–28.3]	.018
Waist circumference (cm)	93.4 ± 1.6	86.3 ± 0.9	.0007*
Hip circumference (cm)	102.5 [99.2–104.5]	99 [98.1–103.2]	.100
WHR	0.9 [0.9–1.0]	0.8 [0.8–0.8]	.085
WHtR	0.6 ± 0.01	0.5 ± 0.007	.013
Subscapular SF (mm)	25.4 [21.7–28.9]	22.3 [21.9–25.5]	.675
Triceps SF (mm)	23.4 [20.3–27.6]	23.4 [21.6–24.9]	.766
Suprailiac SF (mm)	23.9 [20.2–27.2]	22.9 [21.7–24.1]	.981
Medial calf SF (mm)	22.2 [18.4–24.5]	21.3 [20.3–22.5]	.056
Body fat (%)	33.7 ± 0.9	33.1 ± 0.7	.630
Body fat (kg)	22.8 ± 0.9	22.1 ± 0.6	.545
Lean mass (%)	44.6 ± 0.8	66.8 ± 0.7	< .0001*
Lean mass (kg)	44.4 [41.2–47.5]	44.6 [41.8–46.7]	.185
CI	1.3 [1.2–1.3]	1.0 [0.9–1.0]	< .0001*

Data presented as median and interquartile range between [25–75%], Mann-Whitney test. Data presented as media and standard deviation, Unpaired t-student test. BMI: Body mass index; WHR: Waist-to-hip ratio. WHtR: Waist-to-height ratio; CI: Conicity index; SF: Skinfold; kg: Kilograms; m: Meters; cm: Centimeters; mm: Millimeters.

*p<0,05 *versus* Healthy.

**Table 2:** Vital signs during quiet breathing and immediately after maximal voluntary ventilation of the sample (Hypertension vs. Healthy).

Subjetcs (n)	Hypertension (n = 17)	Healthy (n = 17)	*p*	*EZ d’Cohen*	*Power*
**QUIET BREATHING (QB)**					
SBP (mmHg)	130 [130–145]	120 [115–125]	< .0001*	1.77	0.99
DBP (mmHg)	90 [80–100]	80 [80–87.5]	.006*	1.07	0.80
HR (bpm)	78 [73–84]	70 [68–78]	.003*	1.08	0.81
RR (ipm)	18.0 [15.5–22]	15 [14–15.5]	.000*	1.46	0.97
SpO2 (%)	97 [97–98.5]	99 [98–99]	.019*	0.92	0.67
**IMMEDIATELY AFTER MVV**					
SBP (mmHg)	140 [130–150]	120 [120–125]	< .0001*	2.41	0.99
DBP (mmHg)	100 [90–100]	82 [80–89.5]	.000*	1.54	0.98
HR (bpm)	81 [75.5–86.5]	69 [68–77.5]	< .0001*	1.72	0.99
RR (ipm)	17 [15–20]	15 [14–15]	.000*	1.36	0.94
SpO2 (%)	98 [97–98.5]	99 [98–99]	.019*	0.92	0.67

Data presented as median and interquartile range between 25–75%. SBP: Systolic blood pressure; DBP: Diastolic blood pressure; HR: Heart rate; RR: Respiratory rate; SpO2 (%): Peripheral oxygen saturation (percentage); MVV: Maximal voluntary ventilation; ipm: Incursions per minute; bpm: Beats per minute; mmHg: Millimeters of mercury; L/min: Liters per minute

*p<0,05 *versus* Healthy.

The healthy subjects exhibited significantly lower values for waist circumference and conicity index when compared with hypertension individuals. On the other hand, the hypertension individuals presented lower values in percentage of lean mass.

According to BMI, 98.4% of participants were overweight in the hypertension group. Waist circumference suggested that the risk of metabolic complications associated with obesity was increased and substantially increased in 70.5% and 29.4% of participants, respectively. Also, the WHR suggested an increased risk of metabolic complications in fifteen women (> 0.85) and one man (> 0.9), and only 5.8% of participants presented a low risk.

As shown in Table 2, a significant difference was observed regarding SBP, DBP, HR, RR and SpO2 (%) between hypertension individuals and healthy subjects during quiet breathing and immediately after MVV. All the values were lower in the healthy group, except SpO2 (%). According to the effect size and power calculated, the sample of this study showed different behavior at both moments resulted in a large effect on the hemodynamic and respiratory variables, except for SpO2 (%). When compared the intragroup variables (QB *vs*. MVV), no differences were observed in both groups.

Ventilatory capacity measured by maximal voluntary ventilation was significantly lower (MVV = 103.9 ± 23.5 *vs*. 122.6 ± 19, p = .002, L/min) (MVV = 91.1 ± 17.1 *vs*. 107.9 ± 14.7, p = 004, % pred) in hypertension individuals in comparison to healthy subjects.

**Fig 2:**
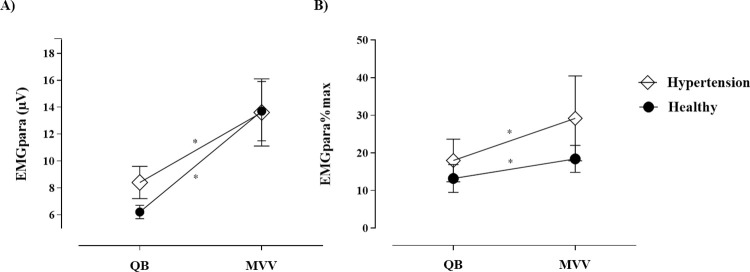
A) Parasternal intercostal muscle activity (EMGpara) expressed as a raw RMS (μV); B) Percentage of parasternal intercostal muscle activity normalized by MIP (EMGpara%max) during quiet breathing (QB) and maximal voluntary ventilation (MVV). Unpaired t-student test, *p<0,05.

Parasternal intercostal muscle’ activity was significantly higher in hypertension individuals in comparison to healthy subjects during quiet breathing. Table 3 summarizes the effect size and power for each NRD variable. Also, percentage of the highest root mean square of EMGpara and neural respiratory drive index were significantly higher in hypertension individuals in comparison to healthy subjects during maximal voluntary ventilation (Fig 2).

**Table 3:** Parasternal intercostal muscle electromyography during quiet breathing and maximal voluntary ventilation of the sample (Hypertension *vs*. Healthy).

Subjetcs (n)	Hypertension (n = 17)	Healthy (n = 17)	*p*	*EZ d’Cohen*	*Power*
**QUIET BREATHING (QB)**					
EMGpara (μV)	8.4 ± 0.3	6.2 ± 0.1	< .0001*	9.83	<0.99
EMGpara%max	17.9 ± 1.3	13.1 ± 0.8	.0066*	4.44	<0.99
NRDi (AU)	320 ± 25	205.7 ± 15	.0004*	5.54	<0.99
**MVV**					
EMGpara (μV)	13.5 ± 0.6	13.8 ± 0.5	.860	0.54	0.3
EMGpara%max	29.3 ± 2.7	18.3 ± 0.8	.000*	5.52	<0.99
NRDi (AU)	3140 ± 259.4	1886 ± 73.1	< .0001*	6.58	<0.99

Data presented as media and standard deviation. EMGpara (μV): Parasternal intercostal muscle electromyography in microvolts; EMGpara%max: Percentage of the highest root mean square of EMGpara during maximal voluntary ventilation; NRDi (AU): Neural respiratory drive index in arbitrary units. Unpaired t-student test

*p<0,05 *versus* Healthy.

**Fig 3:**
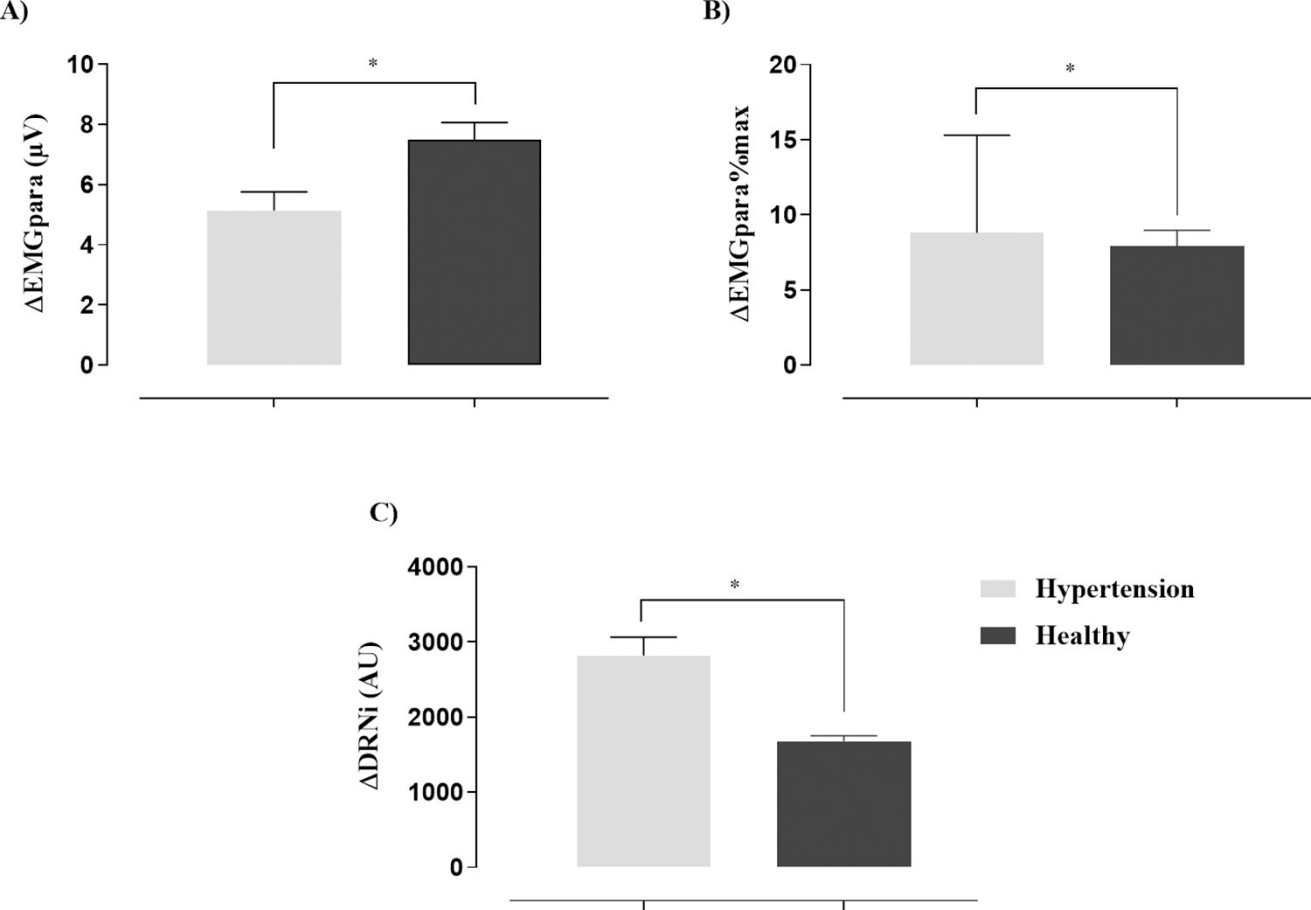
Variation of NRD between QB and MVV of the sample. A) Parasternal intercostal muscle activity (EMGpara) expressed as a raw RMS (μV), *p = .0009, Unpaired t-student test; B) Percentage of parasternal intercostal muscle activity normalized by MIP (EMGpara%max), *p = .0031, Mann-Whitney test; C) Neural inspiratory drive index (NRDi) in arbitrary units (AU), Unpaired t-student test, *p < .0001.

The hypertension group presented greater variation of EMGpara%max and NRDi between QB and MVV, when compared to healthy individuals as shown in Fig 3.

## Discussion

This was the first study that proposed to detail the evaluation of the NRD in individuals with hypertension through EMG of respiratory muscles, as well as to compare them to healthy controls. The main results of the present study were: 1) The NRD and NRDindex during quiet breathing is greater in individuals with hypertension; 2) when required in ventilatory effort maneuvers, the neural respiratory drive increases in order to meet the new cardiorespiratory demands; 3) individuals with hypertension when performing ventilatory effort maneuvers, such as maximal voluntary ventilation, show a greater increase in neural respiratory drive when compared to healthy subjects.

NRD and NRDi measurements using EMG were previously described as non-invasive alternatives to analyze the activity of respiratory muscles and the influence of cardiovascular diseases on respiratory activity [32–34]. An imbalance in the load-to-capacity ratio of the respiratory muscles leads to increased levels of NRD [35]. Studies have described that sympathetic nerve discharge to the cardiovascular system exhibits rhythmic oscillations that are synchronized with the respiratory cycle. Hypertension can disrupt this respiratory–sympathetic modulation since its pathophysiology is characterized by sympathetic overactivity [36].

In the present study, individuals with hypertension presented decreased ventilatory capacity assessed through MVV, corroborating Pramodh et al. (2015) [37]. They observed that individuals with hypertension presented worse pulmonary function (FVC and FEV_1_) than control individuals (80 participants in each group), suggesting that hypertension may cause left ventricular dysfunction [37]. Left ventricular dysfunction increases left atrial pressure and may increase pulmonary artery pressure and interstitial edema in lung. Thus, these conditions decrease FVC and FEV_1_ and reinforce the influence of cardiovascular diseases on increased work of breathing, characterized by the increased NRDi during MVV in the present study.

Macbean et al. (2016) [16] observed a median EMGpara of 4.95 μV (3.35 to 6.93 μV), EMGpara%max of 4.95% (3.39% to 8.65%), and NRDi of 73.62 AU in 63 healthy individuals, which were lower values ​than observed in the present study. Also, respiratory muscle strength (based on maximal inspiratory pressure) was inversely correlated with EMGpara and EMGpara%max in their study [16], corroborating our findings.

Steier et al. (2011) [38] described increased NRD in individuals with controlled and uncontrolled asthma and high variability of the EMGpara overnight, when exacerbation of asthma symptoms usually occurs. Thus, EMGpara may be useful as a non-invasive clinical tool to adjust treatment and detect changes in asthma severity. Increased NRDi was also reported in 30 individuals with chronic obstructive pulmonary disease with acute exacerbations at hospital admission, who presented mean EMGpara of 14.6 μV [30]. About this subject, electrophysiological monitoring allowed the authors to track clinical changes during exacerbation of the disease and predict hospital readmission [30].

Twelve hospitalized individuals with acute exacerbation of cystic fibrosis presented reduced mean EMGpara (-38 ± 19%, p < 0.001) at hospital discharge compared with admission [33]. The reduced NRD was attributed to decreased hyperinflation and changes in the chest wall configuration, and individuals with cystic fibrosis may have an increased load on respiratory muscles due to airway obstruction [33].

Obesity impairs ventilation and increases the work of breathing [35,38], contributing to the onset of respiratory symptoms and a substantial increase in NRD. Individuals with obesity may present 2 to 3-fold higher NRD than individuals without obesity, similar to patients with moderately severe lung diseases. Steier et al. (2009) [38] assessed individuals with and without obesity (30 in each group) using diaphragm EMG and observed that the increased NRD was strongly correlated with BMI (R2 = 0.58; p = 0.001). Also, another study assessed 14 individuals with obesity hypoventilation syndrome (i.e., respiratory failure caused by obesity) and found increased EMGpara (5.5 ± 1.3 μV), EMGpara%max (21.7 ± 8.5%), and NRDi (484.2 ± 214.8 AU) [35].

Macbean et al. (2016) [16] also found no correlation between body fat and EMGpara and EMGpara%max in 63 healthy individuals (31.7% presented overweight and 4.8% obesity). Although attenuated EMGpara in individuals with increased chest wall adiposity was expected, the calculation of EMGpara%max should remain representative of the respiratory load [16] since this measure is normalized using maximal inspiratory pressure. In the present research, the authors took the precaution of having similar characteristics related to weight and BMI, without a significant statistical difference between the groups. Additionally, when tested as confounding factors, BMI and % lean mass did not interfere with NRD during QB. At the time of VVM, the % of lean mass can be a potential confounder for the NRDi.

Individuals with hypertension in this study showed higher values of waist circumference and lower values of lean mass’ percentage corroborating studies showing a correlation between body weight and increased blood pressure, including overweight, obesity, and hypertension [1,23,24]. Also, we observed a high WHR (0.9; Table 1), which was strongly correlated with hypertension [23,24] and indicated high cardiovascular risk among participants. In this regard, further studies need to analyze a sample with different body compositions to improve sensitivity and identify factors influencing the health of this population since increased NRD and obesity were previously associated [34,35].

Increased body fat and reduced lean muscle mass are associated with increased free fatty acids and increased insulin resistance and its consequent relationship with sarcopenia. Furthermore, hypertension is also related to other factors that contribute to muscle fatigue, such as impaired blood flow, reduced delivery, uptake of oxygen and nutrients in the muscle, oxidative stress, physical inactivity, and use of medications. In this aspect, when performing maximal ventilatory efforts, hypertensive patients need to increase NDR to recruit more muscle fibers that optimize ventilation and respond to increased metabolic demand, avoiding fatigue [10,36,37].

Although our study presents some new findings on NRD in individuals with hypertension through EMG of respiratory muscles, some limitations must be reported. The strategies adopted to increase motor unit output when NRD increases are different for the parasternal and diaphragm. The parasternal intercostal muscles have a predominance for motor unit recruitment while the diaphragm shows a predominance for frequency modulation. However, this difference is particularly marked at high levels of diaphragm activation, in patients with chronic airflow limitation or incremental exercise protocols [38–42]. Furthermore, although there is a direct measure of diaphragmatic activity by oesophageal catheters (EMGdi), quantification of NRD using surface recordings of another obligate inspiratory muscle have the advantage over EMGdi recorded using oesophageal catheters of being noninvasive. And several authors show a direct relationship between the measures [43–46].

It is important to make clear that, from the clinical point of view, the neural respiratory drive found in the subjects of the present study should be evaluated and used as an outcome variable in rehabilitation program results. This study was important in detecting that changes in neural respiratory drive may be related to increased respiratory effort, changes in breathing pattern even in the absence of severe impairment of pulmonary function.

## Conclusion

Individuals with hypertension presented higher values of neural respiratory drive during quiet breathing and during a maximal ventilatory ventilation. This increase in the activation of the parasternal intercostal muscles may contribute to a worse efficiency of the respiratory system in the ventilatory demands of this population. And therefore, assessment of neural respiratory drive can be a useful tool in clinical practice.
